# Principal Bioactive Properties of Oleanolic Acid, Its Derivatives, and Analogues

**DOI:** 10.3390/molecules29143291

**Published:** 2024-07-12

**Authors:** Fatin Jannus, Juan Sainz, Fernando J. Reyes-Zurita

**Affiliations:** 1 Department of Biochemistry and Molecular Biology I, Faculty of Sciences, University of Granada, Av. Fuentenueva, 18071 Granada, Spain; jsainz@ugr.es; 2 Genomic Oncology Area, GENYO, Centre for Genomics and Oncological Research, Pfizer/University of Granada/Andalusian Regional Government, Av. de la Ilustración, 114, PTS, 18016 Granada, Spain; 3 Instituto de Investigación Biosanitaria IBs.Granada, 18010 Granada, Spain; 4 Consortium for Biomedical Research in Epidemiology and Public Health (CIBERESP), University of Barcelona, 08908 Barcelona, Spain

**Keywords:** oleanolic acid, anti-inflammatory, antibacterial, antiviral, antidiabetic, hepatoprotective, neuro-protective, anticancer

## Abstract

Natural products have always played an important role in pharmacotherapy, helping to control pathophysiological processes associated with human disease. Thus, natural products such as oleanolic acid (OA), a pentacyclic triterpene that has demonstrated important activities in several disease models, are in high demand. The relevant properties of this compound have motivated re-searchers to search for new analogues and derivatives using the OA as a scaffold to which new functional groups have been added or modifications have been realized. OA and its derivatives have been shown to be effective in the treatment of inflammatory processes, triggered by chronic diseases or bacterial and viral infections. OA and its derivatives have also been found to be effective in diabetic disorders, a group of common endocrine diseases characterized by hyperglycemia that can affect several organs, including the liver and brain. This group of compounds has been reported to exhibit significant bioactivity against cancer processes in vitro and in vivo. In this review, we summarize the bioactive properties of OA and its derivatives as anti-inflammatory, anti-bacterial, antiviral, anti-diabetic, hepatoprotective, neuroprotective, and anticancer agents.

## 1. Introduction

Natural products have historically made an important contribution to pharmacotherapy, particularly for cancer and infectious diseases. They are a traditional system for treating disease throughout the world and have played an essential role in historical and cultural development. The use of these bioactive molecules as medicines dates back thousands of years [1]. It has also been shown that natural products have played a crucial role in the development of modern medicines [2]. Plants have provided the pharmacologically active compounds of many highly successful herbal medicines [3,4]. For example, the Mediterranean diet is healthy because it contains several secondary metabolites, such as the pentacyclic triterpenes OA and maslinic acid (MA), and olive oil is the main source in the diet. It has been associated with a low incidence of several diseases, with promising effects on inflammation, diabetes, cardiovascular diseases, metabolic diseases, several types of cancer, and others [5,6]. The administration of OA incorporated into fatty foods, such as olive oil, contributes to high circulating levels of the compound during the postprandial phase. It has also been shown that the requirement of human serum albumin (HAS) and postprandial triglyceride-rich lipoprotein (TRL), as biological Trojan horse-like carriers of OA, affects the solubility and bio-accessibility of the triterpene and that these mechanisms may be an effective way to deliver OA to target tissues and induce high bioavailability [7].

OA has been isolated from more than 2000 plant species, most of which are medicinal herbs, and foods [8]. OA is abundant in the fruits, leaves, and stem bark of various medicinal plants, including ginseng [9], *Olea europea*, *Calluna vulgaris* [10], *Lantana camara* [11], *Lisgustrum lucidum* [12], and the gape pomace [13]. OA, C30H48O3 (3β-hydroxy-olea-12-en- 28-oic acid), a pentacyclic triterpene, is mainly extracted from the olive plant [14]. OA is often found in the leaves, grains, and fruits of the olive tree in the form of almost pure crystals that protect against fungal attack and act as a defense compound against herbivores or pathogens. In addition to its ecological function in plants, OA has been shown to have important pharmacological activities such as anti-inflammatory, antibacterial, antiviral, anti-diabetic, hepatoprotective, neuroprotective, cardioprotective, and anticancer effects, which have been attributed to OA in various models of disease [13,15,16,17,18,19,20,21].

**Figure 1:**
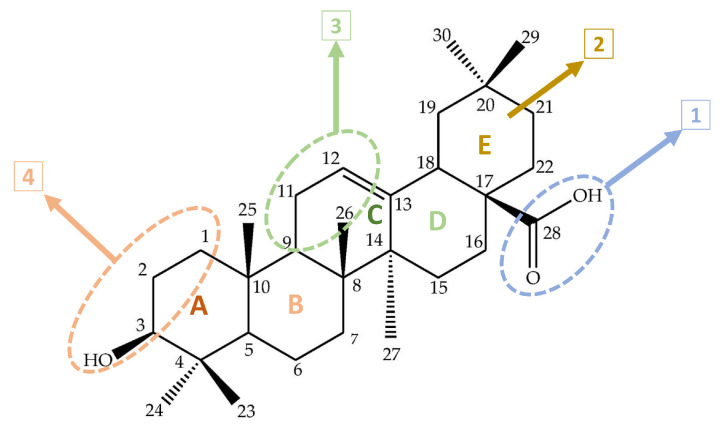
Chemical structure of oleanolic acid. The sites 1, 2, 3, and 4 are the main sites that are modified to improve the bioactivities of oleanolic acid. 1: C-28 modifications; 2: E ring modifications; 3: C ring modifications (C-11 derivatization); 4: A ring modification (C-3, C-2, hydroxyl).

OA is a bioactive and hydrophobic pentacyclic triterpene belonging to the oleanane family, with eight chiral centers. Several chemical modifications have been made to reduce its toxicity and increase its potency, bioavailability, and solubility. The chemical structure of OA consists of five rings of six elements, with a hydroxyl group at carbon C-3; two methyl groups at C-4 and C-20; and one methyl group each at C-8, C-10, and C-14. It also has a carboxyl group at position C-17 and a double bond between C-12 and C-13. The stereochemistry of the -OH group at position C-3 is also important for physiological functions. In addition, the less regular 3α-OH isomers have several biological activities not shared by the more regular 3β-OH isomers. These steric characteristics of the exocyclic methyl and other functions in natural triterpenoids are crucial determinants of their bioactivity and safety in pharmacological use in drug discovery. Additionally, other modifications have been realized to form esters and amides in the A and C rings at the C-2 substitution and at C-28 (Figure 1) [22].

Various efforts have been made to establish the relationship between the structure and functions of OA and its derivatives. This relationship seems to depend on the type of bioactivity studied. For example, in our previous studies, structure–activity relationship (SAR) analysis showed that OA must be conjugated with one or two amino acids and an acyl group to induce HIV-1 inhibition [23]. The anti-influenza activity of OA can be significantly enhanced by conjugating its -COOH or -OH groups with oligosaccharides. On the other hand, the presence of 24 hydroxyl groups induced the inhibition of 5-lipoxygenase (5-LOX) and COX-1 enzymes [24,25,26]. The integrity of the A-ring and the 12-ene moiety appears to be critical for maintaining the enzyme inhibitory activity of PTP1B, where the distance between the acid and the hydrophilic groups played an important role in the activity of these OA derivatives. SAR studies showed that derivatives of OA with structural modifications made in the C-28 carboxyl group, either forming esters or amides, significantly increased their antitumor activity [27,28].

The specific molecular target of OA is related to the type of bioactivity being studied. Depending on each bioactivity, there is a specific molecular target of OA or derivative that is analyzed in each section of the review. However, the transport and distribution of OA in the body is related to serum albumin transport. Due to their amphipathic nature, OA and its derivatives are directly bound to serum albumin, which acts as a carrier [29]. Approximately 98.9 ± 2.5% of circulating OA is bound to human serum proteins (mostly albumin) [30]. It has been described that the binding of OA to albumin can be reversible with respect to pH, which may have an important impact on the distribution of OA in the human body [31,32].

The olive oil industry produces a large volume of solid and liquid waste, which has historically been one of the major problems associated with this industry. As OA and its derivatives can be obtained from two-phase olive mill waste, it is possible to reduce to a large extent the cost of production of OA and its derivatives [33].

Various derivatives of OA have been shown to have a remarkable biological activity. For example, omaveloxolone, a semisynthetic oleanane triterpenoid, is a potent activator of nuclear factor erythroid 2-related factor 2 (Nrf2)-related factor 2, which induces antioxidant function. It was found that the treatment of patients with advanced solid tumors with omaveloxolone showed positive results, as demonstrated by its tolerability by patients and its activation of Nrf2 antioxidant genes [34]. Nrf2 is a key modulator of oxidative stress, and studies support its role in defending against neurodegenerative conditions. Omaveloxolone was recently approved by the FDA as the first treatment for Friedreich’s ataxia (FA), a neurodegenerative disease (NDD) at least in part caused by mitochondrial dysfunction, altered iron metabolism, and the production of reactive oxygen species (ROS) [35]. The development of a new drugs from natural products such as OA, its derivatives, and its analogues represents a potential therapeutic approach that could reduce the adverse effects of conventional drugs. Therefore, several studies, including those conducted by our group, have focused on identifying efficient drug treatments of natural origin by studying the pharmacological effects of OA and its derivatives in different diseases models [13,15,16,17,18,19,20,21].

In this review, we summarize for the first time the in vitro and in vivo studies that were carried out to determine the pharmacological properties of OA, its derivatives, and its analogues, including their roles anti-inflammatory, antibacterial, antiviral, antidiabetic, hepatoprotective, neuroprotective, and anticancer agents.

## 2. Anti-Inflammatory Activity of Oleanolic Acid

Inflammation is a fundamental mechanism that maintains bodily homeostasis. Dysregulation of this process can lead to various diseases. It plays a critical role in recovering from physical injuries and infections. However, uncontrolled acute inflammation can lead to chronic inflammation, contributing to the development of a variety of chronic inflammatory diseases, including cancer, hepatitis, diabetes, and NDDs like Parkinson’s [36,37]. Several factors can induce acute and/or chronic inflammatory responses by causing overproduction of reactive oxygen species (ROS), nitric oxide (NO), and cytokines, which are compounds that initiate and mediate the inflammatory response [38]. Consequently, developing drug therapies that control the inflammatory response is an important advancement in treating these diseases [39]. Anti-inflammatory agents prevent inflammation and can be isolated from natural sources or obtained by chemical synthesis [40,41,42,43,44,45,46,47]. For instance, the evaluation of the anti-inflammatory effects of several aromatic cassane-type diterpenes on a lipopolysaccharide (LPS)-activated RAW 264.7 cell line revealed significant inhibition of NO after treatment [45,46,47].

Several studies have demonstrated the anti-inflammatory effects of OA and its derivatives. It was observed that OA inhibited DSS-induced colitis in Th17 cells by suppressing the expression of IL-1, NF-ĸB, MAPK, and RORγt, as well as by inducing the expression of FOXP3, IL-10, and myeloperoxidase, effectively blocking the colitis process [36]. 11-Oxooleanolic acid derivatives exhibited anti-inflammatory effects in both an LPS-activated BV2 cell inflammation model and a 12-O-tetradecanoyl phorbol-13-acetate-induced ear inflammation mouse model. These derivatives showed stronger anti-inflammatory effects than OA and demonstrated low cytotoxicity. Their anti-inflammatory effect was mediated by the suppression of NO and pro-inflammatory cytokines (IL-1β, IL-6, IL-12, TNF-α, MCP-1, and MIP-1α) and a decrease in anti-inflammatory cytokines such as IL-10 [48].

OA showed a protective effect against *Salmonella typhimurium*-induced diarrhea in BALB/c mice. OA decreased the levels of COX-2 and iNOS and suppressed the secretion of pro-inflammatory cytokines, such as IL-1β, IL-6, and TNF-α. Additionally, OA significantly suppressed the phosphorylation of IκB and reduced the levels of Toll-like receptor 4 (TLR4) and the activation of the MAPK pathway. Therefore, OA can preserve the intestinal tight junction barrier and alleviate diarrhea caused by *Salmonella typhimurium* [49]. Similarly, OA was evaluated in a mouse model by administering it to cardiac α-myosin (MyHc-α614-629)-immunized BALB/c mice in vivo and incorporating it into activated-cardiac cells in vitro. OA treatment markedly reduced disease severity compared to untreated mice. Histological analysis of the heart showed that OA treatment reduced cell infiltration, fibrosis, and dystrophic calcifications. OA also reduced cardiac fibroblast proliferation in vitro and decreased calcium and collagen deposition induced by cytokines relevant to active myocarditis. In addition, in OA-treated experimental autoimmune myositis (EAM) mice, there was a significant increase in the number of Treg cells and the production of IL-10 and IL-35, while pro-inflammatory and profibrotic cytokines were markedly decreased [50]. Furthermore, daily administration of oleanolic acid acetate (OAA) on experimental autoimmune encephalomyelitis (EAE) was induced in C57/BL6 mice using synthesized myelin oligodendrocyte glycoprotein (MOG)35-55 peptide, which significantly increased T-cell proliferation in splenic cells originating from EAE mice. This was accompanied by elevated levels of pro-inflammatory cytokines in the spinal cord and serum protein levels in EAE mice. OAA also increased the secretion of TLR2, suggesting an important therapeutic effect against multiple sclerosis [51].

**Figure 2:**
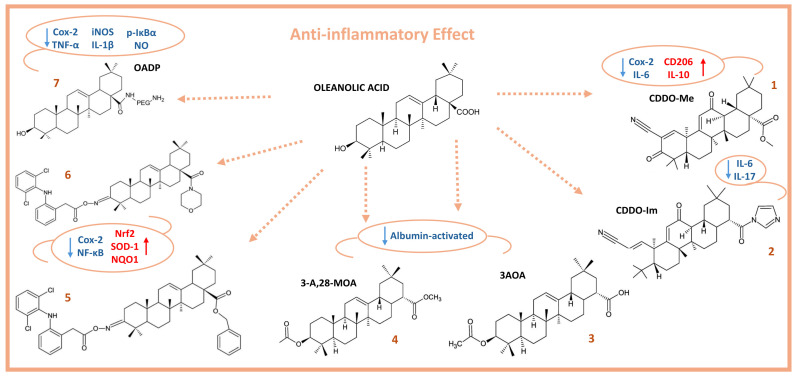
Chemical structure of oleanolic acid and its anti-inflammatory derivatives (red arrows indicate upregulation; blue arrows indicate downregulation).

The derivatives of OA (Figure 2), such as CDDO-Me (**1**), demonstrated anti-inflammatory properties by decreasing the expression of F4/80, CD11c, COX-2, IL-6, KI67, NF-ĸB, and TNF-α, while increasing the expression of CD206 and IL-10 in a rodent model of chronic colon inflammation [52]. Similarly, the synthetic AO derivative CDDO-Im (**2**) blocked the expression of IL-6 and IL-17, thereby alleviating DSS-activated colitis in mice. The acetylated and methylated derivatives of OA, 3-acetoxyoleanolic acid (3-AOA) (**3**) and 3-acetoxy, 28-methylester oleanolic acid (3-A,28-MOA) (**4**), showed an anti-inflammatory effect in serotonin- and fresh-egg-albumin-activated inflammatory models in male Wistar rats weighing 250–300 g. Both semisynthetic products significantly (*p* < 0.05) suppressed albumin-activated inflammation better than OA and indomethacin within 1–5 h after administration. Additionally, both products exhibited membrane-stabilizing effects in a heat-activated hemolysis test, while only 3-AOA showed membrane-stabilizing effects in a hypotonic milieu [53].

A potent anti-inflammatory effect was observed when HepG2 tumor cells were treated with oleanolic acid oxime conjugates with diclofenac. Treatment of transformed human liver epithelial-2 cells (THLE-2), immortalized normal hepatocyte cells, with the conjugates (**5**) (3-diclofenacoxyiminoolean-12-en-28-oic acid benzyl ester) and (**6**) (3-diclofenacoxyiminoolean-12-en-28-oic acid morpholide), induced the expression of Nrf2, superoxide dismutase type 1 (SOD-1), and NAD(P)H quinone dehydrogenase 1 (NQO1). In contrast, the opposite effect was observed in the HepG2 hepatoma cells. These compounds reduced the activation of NF-κB and COX-2 expression in both cell lines [54].

In our previous work, we demonstrated that the diamine-PEGylated oleanolic acid (OADP) (**7**) (Figure 2) has potent anti-inflammatory activity in both in vitro and in vivo models. In LPS-stimulated RAW 264.7 cells, OADP inhibited the expression of TNF-α, IL-1β, iNOS, and COX-2, as well as the production of p-IκBα and NO. In addition, OADP was evaluated in a mouse model of acute ear edema in male BL/6J mice, where it markedly suppressed edema and reduced ear thickness by 14% more than diclofenac [44].

## 3. Antibacterial Activity of Oleanolic Acid

Several infections caused by bacteria, particularly those resistant to antibiotics, represent a global public health challenge. According to the World health organization (WHO), antimicrobial resistance (AMR) is one of the most important global health problems, causing substantial mortality, morbidity, and economic burden [55]. The antibacterial properties of OA have been tested using several bacterial species. Early studies showed that OA has an important antibiotic effect by suppressing the synthesis of insoluble glucosyltransferase (Gtase) in *Streptococcus mutans* [56]. In the context of Mycobacterium tuberculosis, a leading cause of global mortality, OA isolated from *Lantana hispida* exhibited potent activity with a minimum inhibitory concentration (MIC) value of 25 μg/mL [57]. OA demonstrated an MIC of 50 µg/mL against *Mycobacterium tuberculosis* strains resistant to streptomycin, isoniazid, rifampin, and ethambutol. The antibacterial properties of OA have also been evaluated in human bacterial pathogens, including *S. pneumoniae* (MIC of 16 μg/mL), methicillin-susceptible and methicillin-resistant *Staphylococcus aureus* (MIC of 8 μg/mL and 64 μg/mL, respectively) [58], *Bacillus subtilis* (MIC of 8 μg/mL), *B. cereus*, *Enterococcus faecalis* (MIC of 6.25–8 μg/mL), *E. faecium* (MIC of 8 μg/mL), and *Pseudomonas aeruginosa* (MIC of 256 μg/mL) [59,60].

Additionally, previous research has shown that OA is highly active against *E. faecalis*, with a MIC of 6.25 mg/L, and was moderately active against the *S. aureus* strains, which varied in their antibiotic susceptibility pattern. OA also exhibited significant effects against *M. tuberculosis* H37Rv strain [61]. Other studies have shown that *E. coli* treated with OA undergoes modified synthesis of the DnaK protein and experiences a heat shock response [62]. Another article showed that AO suppressed the peptidoglycan turnover in *Listeria monocytogenes*, thereby affecting the amount of muropeptides and the bacterial cell wall [63]. Additionally, OA has been shown to be effective against *L. monocytogenes*, *E. faecium*, and *E. faecalis* (MICs from 16 to 32 μg/mL) by damaging their cell membranes. Moreover, concentrations of OA higher than 128 μg/mL decreased the viability of human epidermoid cancer cell HEp-2 cells [64].

**Figure 3:**
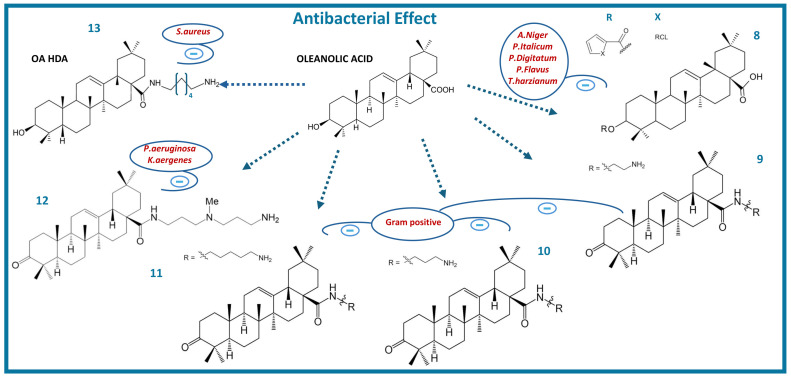
Chemical structure of oleanolic acid and its antibacterial derivatives (blue minus sign indicates growth inhibition).

On the other hand, derivatives of OA obtained by introducing an acyl substituent at the C-3 hydroxyl (Figure 3) gave rise to potent antibacterial agents. Both OA and its acylated analogue have shown antimicrobial activity against two Gram-positive bacteria and two Gram-negative bacteria. One derivative, (3β)-3-((thiophene-2-carbonyl)oxy)-spirost12-en-28-oic acid (**8**), showed effective antibacterial activity against *A. niger*, *P. italicum*, *P. digitatum*, *A. flavus,* and *T. harzianum* [65]. Additionally, the evaluation of antimicrobial effects of a series of oleanolic acid derivatives with oxo- or 3-N-polyamino-3-deoxy-substituents at position C3 and carboxamide function at position C17, along with variable long chain polyamines (Figure 3), showed a good to moderate effect against Gram-positive bacteria such as *Staphylococcus aureus*, *Staphylococcus faecalis,* and *Bacillus cereus*, with MIC values ranging from 3.125 to 200 µg/mL. Furthermore, these products exhibited major antimicrobial effects against Gram-negative bacteria including *E. coli*, *Pseudomonas aeruginosa*, *Salmonella enterica*, and *Enterobacter aerogenes* (EA289). The diamino derivatives (**9**), (**10**), and (**11**) showed efficient antimicrobial activity against Gram-positive bacteria with MICs from 6.25 to 200 µg/mL. However, the compound (**12**) exhibited low MICs against several multidrug-resistant bacteria such as *Pseudomonas aeruginosa* and *Klebsiella aerogenes* EA289. This compound (**12**) disrupts the outer membrane integrity of the Gram-negative bacteria *Pseudomonas aeruginosa* in a manner similar to antimicrobial peptides (AMP) polymyxin B nonapeptide (PMBn) [66].

Another derivative, oleanolic acid-hexane-1,6-diamine (OA-HAD) (**13**), showed significant antimicrobial effect in vitro and decreased the toxicity of the parent compound OA by decreasing MIC in most of the Gram-positive bacteria tested, highlighting its effectiveness against *Staphylococcus aureus* and methicillin-resistant *Staphylococcus aureus* (MRSA). In addition, OA-HAD increased its antimicrobial effect and decreased the MIC50 (MIC value at which growth was inhibited by 50%) against MRSA by 87% (MIC50 of 10 μg/mL) compared to the parent compound OA (MIC50 75 μg/mL) [67]. Further studies demonstrated the efficacy of co-treatment of OA and β-lactam drugs in a mouse infection model, showing an important synergistic effect between the two. The survival rate of mice infected with *S. aureus* and *E. coli* increased from 25.0% to 75.0% following this combination therapy. Co-therapy with OA to target drug-resistant enzymes may be a successful treatment approach for drug-resistant bacterial infections [68].

## 4. Antiviral Activity of Oleanolic Acid

Viral infections pose a serious threat to global public health. There is an urgent need to improve the treatment of human immunodeficiency virus (HIV), influenza, hepatitis, and herpes, which depends on the discovery of new biomedical drugs with low toxicity. Antivirals are compounds that prevent or eliminate viral infections and can be isolated from natural sources or obtained by chemical synthesis. Natural products are one of the main sources for antiviral drug discovery. Most natural triterpenes, such as OA, exhibit prominent antiviral activity [65]. OA has potent antiviral activity against both ACV-sensitive and -resistant HSV-1 strains in different cell types. OA showed an anti-herpes virus effect against herpes simplex virus type 1 (HSV-1) (EC50 = 6.8 g/mL) and HSV-2 (EC50 = 7.8 g/mL). This study revealed that OA exhibits a potent anti-HSV-1 effect in the early stage of infection by dysregulating the viral UL8 protein, a component of the viral helicase-primase complex crucial for viral replication. As a consequence, OA improved the skin lesions in an HSV-1 infection-mediated zosteriform model [16].

**Figure 4:**
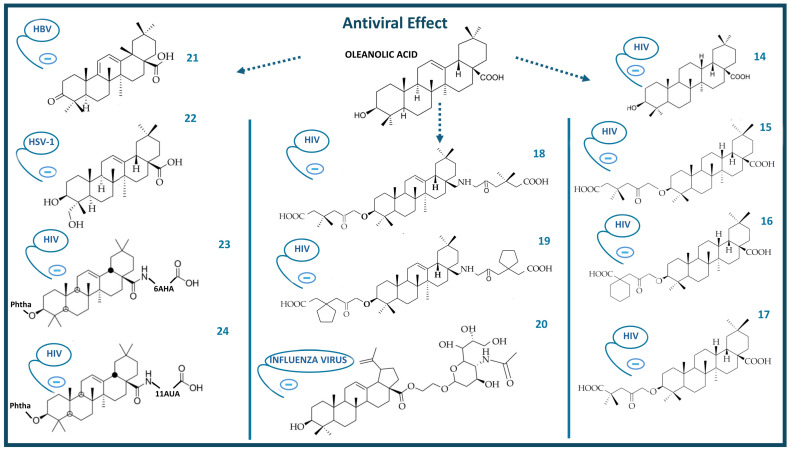
Chemical structure of oleanolic acid and some of its antiviral derivatives (blue minus sign indicates virus inhibition).

New derivatives of OA (Figure 4), with a modified C12-C13 double bond, such as compound (**14**), were three times more active than OA against HIV infection. The esterification of (**14**) produced compounds (**15**), (**16**), and (**17**), which were five times more active than OA, with compound (**17**) showing remarkable activity in HIV infection [69]. Compounds (**18**) and (**19**) were obtained from (**14**) by converting the C28-carboxyl to aminomethyl group. These derivatives were 10 times more active compared to OA against HIV infection [69,70]. The antiviral activity of compound (**20**) was evaluated against the influenza A/WSN/33 (H1N1) virus in Madin–Darby canine kidney (MDCK) cell culture. This compound showed a very potent effect with a half-maximal inhibitory concentration (IC_50_) of 41.2 μM and inhibited the binding of the influenza virus hemagglutinin protein to host cells [71].

Regarding hepatitis, several studies evaluated anti-hepatitis analogues of OA and found that compound (**21**) exerted the highest anti-hepatitis effect in vivo and in vitro by inhibiting HBV DNA replication, blocking hepatitis B surface antigen (HbsAg) secretion and reducing hepatitis B viral protein (HbeAg) secretion [72]. Fifteen oleanane-type triterpenoids were evaluated as anti-herpes simplex virus type 1 (HSV-1) agents. OA and its derivative (**22**) presented moderate anti-HSV-1 activity [73]. In our previous research, we evaluated the antiviral activity of several derivatives of OA against HIV. We showed that the presence of the phthaloyl group at C-3 significantly increased the inhibition of HIV-1 protease, with IC_50_ concentration values below 1 μM in compounds (**23**) and (**24**) represented by 0.79 and 0.88 μM, respectively [23].

## 5. Antidiabetic Activity of Oleanolic Acid

Diabetes is a complex, progressive, and chronic disease resulting from impaired secretion or sensitivity to insulin. Type 2 diabetes mellitus (T2DM) is a common form of diabetes described as hyperglycemia resulting from insulin resistance or insufficient insulin secretion by pancreatic β-cells. There is increasing evidence that T2DM is associated with obesity and the development of multiple diseases, including heart, liver, and kidney disease. It also manifests in various macrovascular/microvascular complications affecting organs such as the arteries, eyes, kidneys, and nerves [74,75]. Great efforts are currently being made to obtain synthetic derivatives as improved antidiabetic drugs, such as iminosugars and sugar derivatives [76,77].

Plant-derived OA alleviated hyperglycemia and reduced hemoglobin A1c (HBA1c) and erythropoietin (EPO) concentrations in STZ-induced diabetic rats. It also significantly increased red blood cell (RBC) count and other red blood cell indices, improved the antioxidant status of RBCs, and reduced oxidative stress [78].

The protective effect of OA is associated with therapeutic memory, as demonstrated by the maintenance of reduced glycemic levels in mice 4 weeks after the end of OA treatment. This therapeutic memory is associated with Forkhead-box-O1 (FOXO-1) acetylation. Additionally, the expressions of histone deacetylase (HDAC) 1 and 2 and glucose 6-phosphatase (G6Pase) were suppressed, while the expression of histone acetyltransferase 1 (HAT1) was increased, suggesting that enzymes involved in epigenetics may play a role in the maintaining glycemic control in T2DM, particularly with OA treatment [79,80].

The anti-diabetic effect of OA has been related to the reduction of ghrelin expression and decreased food intake [81]. Furthermore, OA prevents and reduces insulin resistance induced by Aroclor 1254 treatment in mice; suppresses the increase in ROS; and inhibits the expression of NADPH oxidase (NOX-4), GC-LC, GC-LM, GPX-1, and SOD-1 and SOD-2. These effects are suggested to be mediated by the PPAR-γ signaling pathway through the regulation of hepatocyte nuclear factor 1b [82]. These finding highlight the significant therapeutic effect of OA on insulin resistance. In other work, it was shown that after treatment of C2C12 cells with the ethyl acetate (EtOAc) extract and OA at a dosage of 3 μg/mL for 48 h resulted in a significant increase in the mitochondrial activities in vitro of cultured C2C12 myoblast cells. Additionally, it was shown that OA modulates glucose uptake through stimulation of glucose transporter (GLUT) [13].

**Figure 5:**
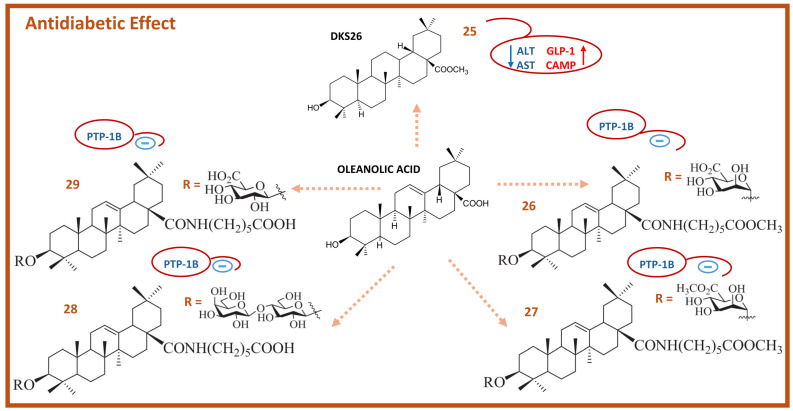
Chemical structure of OA and some of its antidiabetic derivatives (red arrows indicate up-regulation; blue arrows indicate down-regulation; blue minus sign indicates inhibition).

OA derivatives have also shown significant antidiabetic effects (Figure 5). For example, 12,13 DKS26 (**25**) is a hypoglycemic therapeutic agent that reduces plasma glucose levels and glycosylated serum proteins, as well as alanine aminotransferase (ALT) and aspartate aminotransferase (AST). This compound also improves plasma lipid profiles, increases plasma insulin levels, and enhances glucagon-like peptide-1 (GLP-1) through the cyclic adenosine monophosphate (cAMP) and phospho-PKA cascade [83]. Another OA derivative, 2α, 3β, 23α, 29α tetrahydroxyoleano-12(13)-en-28-oic acid, extracted from the aerial parts of *Malva parviflora*, has shown an antidiabetic effect in streptozotocin (STZ)-nicotinamide-induced diabetes type 1 and 2 in mice. This derivative regulates glucose metabolism, liver glycogen, lipid peroxidation, lipid profile, body weight gain, and glucokinase and hexokinase activities, as well as inhibiting triglycerides, total cholesterol, and low-density lipoprotein levels. It also reduces serum glutamic-oxaloacetic transaminase (SGOT), serum glutamic pyruvic transaminase (SGPT), Src-like adaptor protein (SALP), and glucose-6-phosphatase levels [84].

Other synthetic OA derivatives, such as (**26**) (3β-O-α-d-mannuronopyranoside), (**27**) (3β-O-α-d-mannuronopyranosyl-6-methyl ester), (**28**) (3β-O-β-D-lactopyranoside), and (**29**) (3β-O-β-D-glucuronopyranoside), have demonstrated inhibitory activity against protein tyrosine phosphatase 1B (PTP-1B), which is involved in insulin resistance. These derivatives (Figure 5) exhibit potent inhibitory activities with IC_50_ values of 1.91, 12.2, 9.21, and 0.56 μM against PTP-1B, respectively. They also achieve high percentage inhibitions of 98.60%, 81.88%, 83.72%, and 71.99%, respectively. Moreover, compounds (**26**) and (**29**) have shown promising insulin-sensitizing effects [85,86].

## 6. Hepatoprotective Activity of Oleanolic Acid

**Figure 6:**
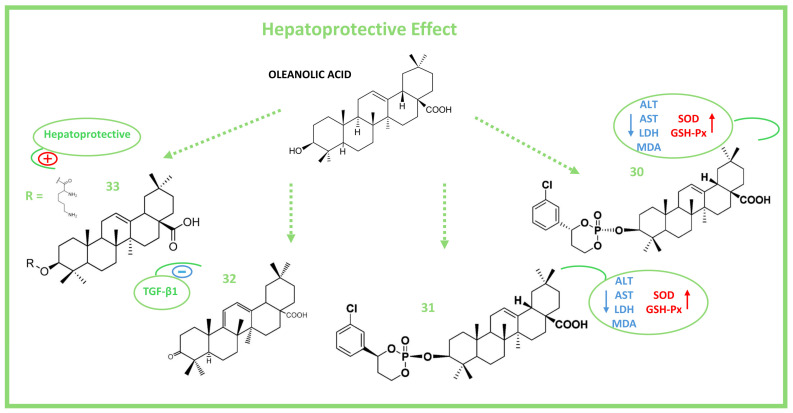
Chemical structure of OA and some of its hepatoprotective derivatives (red arrows indicate up-regulation; blue arrows indicate down-regulation; red plus sign indicates activation; blue minus sign indicates inhibition).

One of the most important pharmacological proprieties of OA and its derivatives is hepatoprotection. OA has demonstrated hepatoprotective effects by modulating the activities of several CYP P450 enzymes in human liver microsomes (Figure 6) [87].

Alcoholic liver disease (ALD) is indeed a major cause of death, and oxidative stress has been described as an important factor in the damage caused by this disease. OA prevented alcohol-induced oxidative injury by down-regulating serum AST, ALT, and ATP levels, reducing hepatic SOD and chloramphenicol acetyltransferase (CAT) activities and increasing GSH levels. These protective effects of OA were associated with the activation of antioxidant pathways NRF2, heme oxygenase-1 (HO-1), and SOD-1, and glutathione reductase (GR) expression, as well as the suppression of pro-inflammatory cytokines, such as TNF-α and IL-6 [88]. Furthermore, in an obesity-related non-alcoholic fatty liver disease (NAFLD) model using obese rats fed with a high-fat diet (HFD), OA restored intestinal barrier function disrupted by the diet and inhibited endotoxin-mediated Toll-like receptor 4-related pathways. It also mitigated endotoxemia and systemic inflammation, and it balanced gut–liver axis homeostasis [89].

OA inhibits the enzyme CYP2E1, which produces toxic aldehydes and free radicals during ethanol metabolism by the inducing of Nrf2 and activating Takeda G-protein-coupled receptor (TGR5) in the liver [87]. Thus, OA derivatives, such as CDDO-Im and CDDO-Me, are potent Nrf2 activators [17]. The OA derivatives (Figure 6) (**30**) (cis-3-O-[4-(S)-(3-chlorophenyl)-2-oxo-1,3,2-dioxa-phosphorinan-2-yl]oleanolic acid) and (**31**) (cis-3-O-[4-(R)-(3-chlorophenyl)-2-oxo-1,3,2-dioxaphosphorinan-2-yl]-oleanolic acid) have demonstrated hepatoprotective effects against CCl4-induced liver injury in mice. In vivo treatment of mice with (**30**) and (**31**) at a dose of 15 mg kg^–1^ resulted in a significant increase in levels of ALT, AST, LDH, and MDA and increased SOD and glutathione peroxidase (GSH-PX) activities compared with the control group [90].

On the other hand, the OA derivative Oxy-Di-OA (**32**) (D50 = 714.83 mg/kg) has exhibited protective effects against CCl4-stimulated liver injury in rats by decreasing serum levels of AST and ALT and inhibiting the expression of transforming growth factor-β1 (TGF-β1) [91]. Novel OA aminoacyl derivatives synthesized at C3, such as the OA-lysine derivative (**33**), have demonstrated greater hepatoprotective properties than OA itself against acute CCl4-induced liver injury in mice [92].

## 7. Neuroprotective Activity of Oleanolic Acid

Neurodegenerative diseases are characterized by complex pathophysiological changes in the brain, often exacerbated by the blood–brain barrier (BBB), which restrict the entry approximately 99% of foreign substances from entering the brain [93]. Despite these challenges, the neuroprotective effects of OA and its derivatives have been extensively studied, showing promising therapeutic potential across various neurological conditions. OA has been found to minimize brain damage in the short-term and long-term in an ischemia–reperfusion mouse model. It also has a neuroprotective effect in several brain pathologies such as Parkinson’s disease and ischemic damage [94,95].

Moreover, OA and its derivatives have shown neuroprotective effects in several in vivo models, including those induced by hydroxydopamine, Aβ25-35 injection, Alzheimer’s disease, and stroke. Notably, OA has been observed to induce migration and proliferation of neural stem cells (NSCs) and promote their differentiation into neurons rather than glial cells. This effect was mediated by increased expression of microtubule-associated protein-2 (MAP-2) and the proneural transcription factor Ascl1 (Mash1) and reduced expression of glial astrocyte-specific markers such as fibrillary acidic protein and nestin. In addition, OA was able to activate the expression of GSK-3β phosphorylated and β-catenin [96,97].

A DNA microarray study found that OA differentially regulates 183 genes, among which 87 are associated with the transcription factor NKX-2.5. These results demonstrated that OA induces NSC differentiation into neurons through NKX-2.5-related components [98]. Furthermore, it has been described that OA and its derivatives induced neural differentiation and synapse plasticity through the HDAC5 phosphorylation pathway [99]. Oral supplementation of OA in male Wistar rats treated with sodium fluoride resulted in an improvement in protein and nucleic acid content, proteolytic enzyme activities, and other parameters of oxidative stress [100].

Combining L-dopa with OA treatment attenuated the side effects of L-dopa in Parkinson’s disease, reducing the asymmetry in limb movement induced by the unilateral injection of 6-hydroxydopamine (6-OHDA) [101]. OA treatment in depression-like mice (long-term corticosterone treatment) decreased serine/threonine protein kinase 1 (SGK1) levels and induced the brain-derived neurotrophic factor BDNF-AKT/mTOR pathway, increased sucrose preference, and decreased immobility time in the animals [102].

Pretreatment with OA reduced ischemic injury in a middle cerebral artery occlusion (MCAO) mouse model, as well as the cerebral infarct area and neurological symptom score at 24 h postinjury. After 9 weeks of ischemic injury, OA reduced neurological deficits, inhibited astrocyte proliferation, and stimulated the expression of synapse-related proteins and double cortin-expressing (DCX+) cells in the hippocampus [94]. OA reduced brain damage in acute and chronic ischemic stroke in the MCAO model, including neuronal apoptosis, and decreased the expression of the NLRP3 inflammasome in microglia [103]. Therefore, these results suggest that OA has significant neuroprotective properties, making it an effective and important therapeutic agent in the treatment of neurodegenerative diseases.

## 8. Anticancer Activity of Oleanolic Acid

According to the WHO, cancer is the leading cause of death worldwide. It comprises a group of complex diseases for which the search for effective therapies is crucial in the absence of a definitive or clear therapeutic solution [104]. In cancer, the homeostatic balance between proliferation and cell death is lost. Apoptosis, a physiological process of cellular autoregulation, is one of the main mechanisms controlling this balance [105]. The anti-proliferative effect of OA has been confirmed on several cancer cell lines. OA activates apoptosis in the hepatocellular carcinoma (HCC) cells SMMC-7721 and BEL-7404 by altering the Bax/Bcl2 balance, inducing the release of cytochrome-c and activating caspase-9 and caspase-3 [106,107]. In four human liver cancer lines (HepG2, Hep3B, Huh7, and HA22T), OA activated apoptosis by increasing caspase-8 and caspase-3 [108]. Therefore, it is an essential means of cancer progression, and OA treatment significantly reduced intratumoral microvessel density (MVD) in colorectal cancer (CRC) mice and inhibited tumor growth [109].

**Figure 7:**
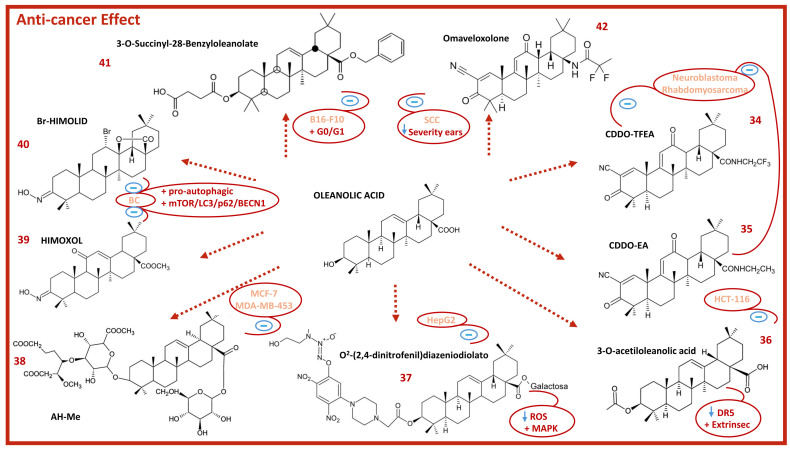
Chemical structure of OA and some of its anticancer derivatives (blue arrows indicate down-regulation; red plus sign indicates activation; blue minus sign indicates inhibition).

OA derivatives (Figure 7) have also shown important anticancer and proapoptotic effects. The OA derivative CDDO-Me (**1**) (Figure 2) inhibited Janus kinase (JAK) and signal transducer and activator of transcription 3 (STAT3) pathways in MDA-MB-468 breast cancer cells [110]. In breast cancer cells, the OA derivative CDDO-Im (**2**) (Figure 2) in combination with the Gemini vitamin D analogue, ABXL0124, reduced cell proliferation and the levels of the proteins HER2, receptor tyrosine kinase 2 (Erb-B2), phosphorylated extracellular signal-regulated kinase 1/2 (pErk1/2), phosphorylated protein kinase B (pAKT), c-Myc, cyclin D1, and Bcl-2 [111]. CDDO-Im has been shown to be a potent inhibitor of the epidermal growth factor receptor (EGFR) and STAT3/SRY-Box transcription factor 2 (Sox-2) signaling pathways in tumor-inducing macrophage (TIM) cells, which stimulate breast cancer growth and metastasis [112]. CDDO-Me and CDDO-Im suppressed the growth of glioblastoma (U87MG, U251MG) and neuroblastoma (SK-N-MC) cells, showed potent anticancer activity, and activated apoptosis in these cell lines [113]. CDDO, CDDO-Me, CDDO-Im, CDDO-TFEA (**34**), and CDDO-EA (**35**) showed anticancer activity in 22 pediatric solid tumor cell lines, including neuroblastoma, rhabdomyosarcoma, osteosarcoma, and Ewing’s sarcoma. In addition, CDDO-Me induced apoptosis through mitochondrial disruption and activation of caspase-3 and caspase-8 [114]. The 3-O-acetyloleanolic acid (**36**) induced apoptosis in HCT-116 cells through the activation of the extrinsic apoptotic pathway by increasing the expression of death receptor 5 (DR5) [115]. O^2^-(2,4-Dinitrophenyl) diazeniodiolate (**37**) showed potent antitumoral activity in vitro and in vivo, inducing apoptosis in HepG2 cells, causing cell cycle arrest at the G2/M phase, activating both the mitochondrial and MAPK pathways, and decreasing intracellular ROS production [116]. The OA derivative, methyl ester achyranthoside H (AH-Me) (**38**) showed remarkable cytotoxicity in human MCF-7 and MDA-MB-453 breast cancer cell lines, inducing apoptosis by caspase-3 activation [117].

The compounds obtained by conjugation of diclofenac with the novel oleanolic acid oximes (OAO) morpholide and benzyl ester (4d, 3-diclofenacoxyiminoolean-12-en-28-oic acid morpholide) and (4c, 3-diclofenacoxyiminoolean-12-en-28-oic acid benzyl ester) showed an important anticancer activity in HCC, while compounds (**5**) and (**6**) (Figure 2) decreased activation of Nrf2 and induced apoptosis. The conjugates also increased ROS production in HepG2 cell lines. Finally, these conjugates reduced tumor volume in mouse xenografts [41]. In a previous study, we demonstrated that OADP compound (**7**), an OA derivative (Figure 2), has a potent antitumor effect in HCC, with IC_50_ = 0.14 µg/mL. Treatment of HepG2 cells with OADP-induced cell cycle arrest in the G0/G1 phase and the loss of mitochondrial membrane potential (MMP). Moreover, OADP induced the activation of extrinsic and secondary activation of the intrinsic apoptotic pathways through the upregulation of caspase-8, caspase-9, caspase-3, Bak, p21, and p53, accompanied by the downregulation of Bcl-2 [118].

The two semisynthetic derivatives of OA (Figure 7), methyl 3-hydroxyimino-11-oxoolean-12-en-28-oate (HIMOXOL) (**39**) and 12α-bromo-3-hydroxyimonoolean-28→13-olide (Br-HIMOLID) (**40**), showed antiproliferative effects in breast cancer cells (BC). Both showed a pro-autophagic effect mediated by the mTOR/LC3/p62/BECN1 signaling pathway and reduced the migration of HER2-positive SK-BR-3 breast cancer cells through the integrin β1/FAK/paxillin pathway [119]. Our group also reported that the derivative 3-O-succinyl-28-O-benzyl oleanolate (**41**) showed higher cytotoxicity in the treatment of B16–F10 melanoma cells by inducing strong G0/G1 cell cycle arrest and resulting in between 72–95% apoptosis [120]. In a non-Herlitz, junctional epidermolysis bullosa (nH JEB) mouse model, the OA derivative omaveloxolone (**42**) significantly decreased phenotypic severity in the affected ears and the viability of squamous cell carcinoma (SCC) cells [121].

## 9. Conclusions

In conclusion, plant-derived OA and its derivatives have been shown to be excellent compounds with diverse pharmacological properties such as anti-inflammatory, antibacterial, antiviral, anti-diabetic, hepatoprotective, neuroprotective, and anticancer effects in various models in vitro and in vivo, due to their amphipathy, bioavailability, and potency. Some of its derivatives are found in clinical trials. Therefore, the anti-inflammatory effects of OA and its derivatives are mainly regulated by the expression of pro-inflammatory cytokines (IL-1β, IL-6, IL-12, TNF-α) and lipid mediators (COX-1, COX-2), reactive oxygen species, and nitric oxide through the activation of the MAPK pathway. In addition, the antibacterial activity of OA and its derivatives demonstrated an effective antibiotic effect on several Gram-negative and Gram-positive bacteria. Furthermore, the antiviral properties of OA, its derivatives, and analogues exhibited a remarkable antiviral effect on a wide range of viral species. Furthermore, OA and its derivatives showed antidiabetic activity in several models by suppressing the expression of HDACs 1, HDACs 2, G6Pase, ROS, NADPH, NOX-4, GPX-1, SOD-1, and SOD-2, as well as by increasing the expression of plasma insulin levels, GLP-1, and HAT1. In addition, OA and its derivatives have hepatoprotective properties by down-regulating serum AST, ALT, and ATP levels. OA derivatives also increased GSH, ALT, and LDH levels, as well as SOD and GSH-PX activities. Moreover, the neuroprotective effect of OA was enhanced by migration and proliferation of NSCs. Differentiation was induced by increased expression of MAP-2. Its derivative reduces the side effects of L-dopa in Parkinson’s disease. Additionally, the anticancer properties of OA and its derivatives have demonstrated an important anticancer effect in various models in vitro and in vivo. This review highlights the most important biological properties of OA, its derivatives, and analogues. OA derivatives and analogues represent a potential alternative therapy in the treatment of all these diseases.

## Data Availability

Data are contained within the article.

## Abbreviations

3-A,28-MOA	3-Acetoxy, 28-methylester oleanolic acid
3-AOA	3-Acetoxyoleanolic acid
5-LOX	5-Lipoxygenase
6-OHDA	6-Hydroxydopamine
AH-Me	Methyl ester achyranthoside H
AKT	Alpha serine/threonine-protein kinase
ALD	Alcoholic liver disease
ALT	Alanine aminotransferase
AMP	Antimicrobial peptides
AMR	Antimicrobial resistance
AST	Aspartate aminotransferase
ATP	Adenosine triphosphate
Bax	Bcl-2 associated X protein
Bak	Bcl-2 homologous antagonist killer
BC	Breast cancer
Bcl2	B-cell lymphoma 2
BECN1	Beclin-1
BBB	Blood–brain barrier
Br-HIMOLID	12α-Bromo-3-hydroxyimonoolean-28→13-olide
cAMP	Cyclic adenosine monophosphate
CAT	Chloramphenicol acetyltransferase
CD206	Cluster of differentiation 206
CDDO	2-Cyano-3,12-dioxoolean-1,9-dien-28-oic
CDDO-Im	CDDO imidazolide
CDDO-EA	CDDO ethyl amide
CDDO-Me	CDDO methyl ester
CDDO-TFEA	CDDO-trifluoethyl amide
CNS	Central nervous system
COX	Cyclooxygenase
CRC	Colorectal cancer
CYP	Cytochrome
DCX	Doublecortin
DKS26	DihydroOA methyl ester
DR5	Death receptor 5
EAE	Experimental autoimmune encephalomyelitis
EAM	Experimental autoimmune myositis
EGFR	Epidermal growth factor receptor
EPO	Erythropoietin
Erb-B2	Receptor tyrosine kinase 2
Erk1/2	Extracellular-signal-regulated kinase 1/2
EtOAc	Ethyl acetate
FA	Friedreich ataxia
FAK	Focal adhesion kinase
FDA	Food and drug administration
FOXO-1	Forkhead-box-O1
FOXP3	Forkhead box P3
G6Pase	Glucose 6-phosphatase
GC-LC	Glutamate cysteine ligase proteins
GC-LM	Glutamate-cysteine ligase
GLP-1	Glucagon-like peptide-1
GLUT	Glucose transporter
GPX-1	Glutathione peroxidase 1
GR	Glutathione reductase
GSH	Glutathione
GSH-PX/GPx	Glutathione peroxidase.
GSK-3β	Glycogen synthase kinase-3 beta
Gtase	Glucosyltransferase
HAS	Human serum albumin
HAT1	Histone acetyltransferase 1
HbA1c	Hemoglobin A1c
HBeAg	Hepatitis B viral protein
HBsAg	Hepatitis B surface antigen
HCC	Hepatocellular carcinoma
HDAC	Histone deacetylase
HER2	Human epidermal growth factor receptor 2
HFD	High-fat diet
HIMOXOL	Methyl 3-hydroxyimino-11-oxoolean-12-en-28-oate
HIV	Human immunodeficiency virus
HO-1	Heme oxygenase-1
HSV	Herpes simplex virus
IL	Interleukin
iNOS	Inducible nitric oxide synthase
IκBα	NF-κB inhibitor α
IKK	IkappaB kinase
JAK	Janus kinase
LDH	Lactate dehydrogenase
LPS	Lipopolysaccharide
LC3	Microtubule-associated protein 1A/1B-light chain 3
MA	Maslinic acid
MAP-2	Microtubule-associated protein-2
MAPK	Mitogen-activated protein kinases
MCAO	Middle cerebral artery occlusion
MCP-1	Monocyte chemoattractant protein-1
MDA	Malondialdehyde
MDCK	Madin–Darby canine kidney
MIC	Minimum inhibitory concentration
MIC50	MIC value at which growth was inhibited in 50%
MIP-1α	Macrophage inflammatory protein-1alpha
MMP	Mitochondrial membrane potential
MRSA	Methicillin-resistant staphylococcus aureus
MVD	Microvessel density
NADPH	Nicotinamide adenine dinucleotide phosphate hydrogen
NAFLD	Non-alcoholic fatty liver disease
NDD	Neurodegenerative disease
NF-ĸB	Nuclear factor-kappa B
nH JEB	Non-Herlitz, junctional epidermolysis bullosa
NLRP3	NOD-like receptor family, pyrin domain containing 3
NOX-4	NADPH oxidase 4
NQO1	NAD(P)H quinone dehydrogenase 1
Nrf2	Nuclear factor erythroid 2-related factor 2
NSCs	Neural stem cells
OA	Oleanolic acid
OAA	Oleanolic acid acetate
OADP	Diamine-PEGylated oleanolic acid
OA-HDA	Oleanolic acid-hexane-1,6-diamine
OAO	Oleanolic acid oximes
p21	Cyclin-dependent kinase inhibitory protein-1
p53	Tumor suppressor protein
p62/SQSTM1	Sequestosome-1
PMBn	Polymyxin B nonapeptide
PPAR-γ	Peroxisome proliferator activated receptor γ
PTP-1B	Protein tyrosine phosphatase 1B
RBCs	Red blood cells
RORγt	Retinoic-acid-related orphan receptors gamma
ROS	Reactive oxygen species
SALP	Src-like adaptor protein
SAR	Structure–activity relationship
SCC	Squamous cell carcinoma
SGK1	Serine/threonine protein kinase 1
SGOT	Serum glutamic-oxaloacetic transaminase
SGPT	Serum glutamic pyruvic transaminase
SOD	Superoxide dismutase type
Sox-2	SRY-Box transcription factor 2
STAT3	Signal transducer and activator of transcription 3
STZ	Streptozotocin
T2DM	Type 2 diabetes mellitus
TGF-β1	Transforming growth factor β1
TGR5	Takeda G-protein-coupled receptor
THLE-2	Transformed human liver epithelial-2
TIM	Tumor-inducing macrophages
TLR	Toll-like receptor
TNF-α	Tumor necrosis factor alpha
TOR	Target of rapamycin
TRL	Triglyceride-rich lipoprotein
